# Particle-Size-Sieved Fractions of *Caesalpinia pulcherrima* Seed Flour: Physicochemical, Nutritional, and Functional Properties

**DOI:** 10.1021/acsomega.5c12402

**Published:** 2026-03-30

**Authors:** Poliana Brito de Sousa, Elenilson Godoy Alves Filho, Lorena Mara A. Silva, Neilane Gomes da Rocha, Camila de Carvalho Chaves, Ana Karoline Nogueira Freitas, Heldeney Rodrigues de Sousa, Luis José Duarte Franco, Rafael Audino Zambelli

**Affiliations:** a Departamento de Engenharia de Alimentos, 28121 Universidade Federal do Ceará, Fortaleza 60020-181, Brazil; b Embrapa Agroindústria Tropical, 28121 Universidade Federal do Ceará, Fortaleza 60020-181, Brazil; c Departamento de Tecnologia de Alimentos, Instituto Federal do Maranhão, São Luís 65095-460, Brazil; d Departamento de Química, Instituto Federal do Piauí, Teresina 64000-040, Brazil; e Laboratório de Bromatologia, 592446 Embrapa Meio-Norte, Teresina 64000-040, Brazil

## Abstract

Flamboyant-mirim seed flour (FSF) emerges as a promising natural ingredient obtained from grains rich in proteins, carbohydrates, minerals, and bioactive compounds. This study aimed to characterize FSF and evaluate the effect of particle size on its chemical, physicochemical, technological, mineral, and functional properties. The seeds were milled using a knife grinder, and the flour fractions were separated through sieves of 0.710, 0.500, 0.355, and 0.250 mm. Analyses included proximate composition, technological, and functional parameters, and ^1^H NMR spectroscopy was performed only for the selected 0.250 mm fraction (chosen due to its superior proximate composition) to assess solvent-dependent compositional variability. Data were acquired through triplicate sampling and statistically treated using ANOVA, Tukey’s test, and supervised multivariate analysis (PLS-DA). All analyses were performed in triplicate (*n* = 3), and PLS-DA was validated by Venetian Blinds cross-validation. The 0.250 mm fraction showed higher ash, lipid, protein, fiber, and mineral contents as well as elevated water solubility index and superior oil absorption, foaming, and emulsifying capacities. The 0.500 mm fraction exhibited the highest total phenolic content and antioxidant activity. PLS-DA analysis indicated that pH, soluble solids, color parameters, phenolics, and antioxidant capacity were the main discriminant variables among the samples. Overall, the results demonstrate the potential of FSF as a functional ingredient for innovative formulations in the food industry.

## Graphical abstract

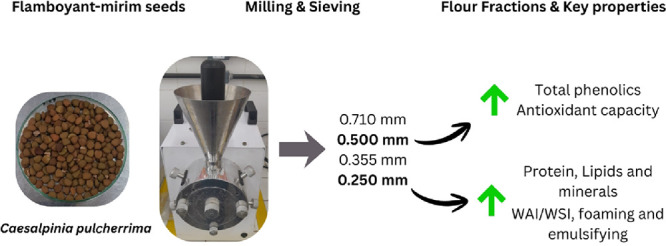

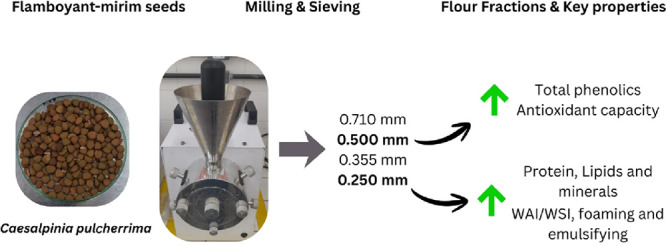

## 1 Introduction

The search for new nutrient-dense food sources is an important strategy to address food insecurity and promote access to healthy and sustainable diets worldwide.^1^ Brazil, one of the most biodiverse countries in the world, harbors an extensive variety of edible plant species distributed across its biomes.^2^ Among these species, *Caesalpinia pulcherrima* (L.) Swartz, commonly known as the flamboyant-mirim or peacock flower, stands out for its ecological adaptability. Although native to Central America, this leguminous plant is well established in several tropical regions, including the Brazilian Caatinga.^3^

From a food-use perspective, it is important to provide an evidence-based rationale for edibility and safety when proposing nonconventional legume seeds as ingredient sources. In general, legume seeds may contain antinutritional factors such as phytates, tannins/polyphenols (often concentrated in seed coats), and protease inhibitors (trypsin inhibitors), which can reduce mineral bioavailability and protein digestibility; however, these compounds are commonly mitigated by processing operations including dehulling and thermal treatments.^4–6^ Seed coats, in particular, are recognized as fiber- and mineral-rich tissues, and their relative contribution to flour fractions can influence the overall nutritional profile.^7^ For *Caesalpinia pulcherrima*, processed seed flours have been previously investigated, and dehulling/thermal processing has been reported to reduce antinutritional factors while supporting the feasibility of using the flour as a food ingredient. In addition, the literature frequently reports composition for whole seeds or dehulled kernels (“seed nuts”), which reinforces that anatomical partitioning and processing steps affect nutrient distribution and direct comparability with sieved flour fractions.^8^

Pharmacological investigations of *C. pulcherrima* have demonstrated that extracts obtained from different plant parts (seeds, flowers, leaves, bark, pods, and roots) using various solvents (aqueous, ethanolic, methanolic, hexanic, chloroformic, or acetonic) exhibit a broad spectrum of biological activities, such as antimicrobial,^9^ antiviral,^10^ antioxidant,^11^ anti-inflammatory,^12^ antiulcer,^13^ anticonvulsant,^14^ and anthelmintic effects.^15^

The seeds of *C. pulcherrima* have been reported to contain proteins, carbohydrates, and essential minerals.^16^ Together with the fruit pericarp, they have also been associated with antimicrobial and antioxidant potential.^9^ Notably, the seed endosperm is described in the literature as a source of galactomannans, polysaccharides widely used as hydrocolloids and dietary-fiber ingredients in food systems.^17^ Although galactomannan content and rheological/gelling behavior were not directly quantified in the present study, our functional tests (water solubility index, oil absorption, foaming, and emulsifying properties) provide an applied assessment of ingredient performance across particle-size-sieved flour fractions. Recent studies have explored *C. pulcherrima* galactomannan-rich materials in edible films, ice creams, and dairy desserts, supporting their relevance for food applications.^18–20^

*Caesalpinia pulcherrima* (L.) Sw. has a long history of use in traditional medicine in different regions. Ethnobotanical records describe the use of decoctions/infusions prepared from the bark, leaves, roots, flowers, or seeds as febrifuge and purgative preparations, for gynecological purposes (emmenagogue), and for gastrointestinal and respiratory complaints such as diarrhea/dysentery, gastritis, bronchitis, and fever.^13,21^ In addition to medicinal uses, reports also indicate that immature pods, seeds, and flowers may be consumed as food in some contexts, supporting the edible potential of this species. From a food-ingredient standpoint, the seeds have been processed into flour, showing relevant nutritional composition, and dehulling/thermal treatments have been reported to reduce antinutritional factors, supporting feasibility for incorporation into formulated foods.^22^ Moreover, the seed endosperm contains a galactomannan with clear technofunctional potential: partial hydrolysis yields a low-viscosity, high-fiber ingredient suitable for liquid foods and its application has been associated with synergistic interactions with starch/milk proteins in dairy desserts, improving viscosity and gel strength.^20,23^

Vegetable flours derived from different grains and seeds have gained increasing attention due to their nutritional value and potential health benefits. They are commonly incorporated into bakery and confectionery products, such as bread, cakes, pasta, and biscuits, to enhance fiber, protein, vitamin, and mineral contents.^24,25^ The particle size of these flours is a critical factor that influences their physicochemical and functional properties, being directly affected by processing operations such as milling, sieving, and mixing.^26^ Controlling particle size distribution through sieving can yield more homogeneous samples, improving hydration capacity, texture, and overall functional performance in formulated foods.^27^

Therefore, this study aimed to evaluate the effect of particle size on the physicochemical, proximate, technological, functional, and mineral properties of flamboyant-mirim seed flour (FSF) fractions obtained by sieve-based size separation. In addition, ^1^H NMR spectroscopy was applied to the selected 0.250 mm fraction (chosen based on its superior proximate composition) to investigate solvent-dependent compositional variability.

## 2 Materials and Methods

### 2.1 Preparation and Fractionation of the Seed Flour

Pods containing seeds of *Caesalpinia pulcherrima* (L.) Swartz (SisGen Registration Code: ABF331B) were collected from the Federal University of Ceará – Pici Campus, Fortaleza, Ceará, Brazil (Latitude: 3°44′38.6″ S; Longitude: 38°34′47.3″W). Pods were harvested from 10 adult plants distributed across the campus. After collection, the seeds from all plants were combined and thoroughly homogenized to form a single composite batch prior to milling and sieve fractionation. Therefore, the study relied on one biological lot (composite batch), and all reported replicates correspond to the analytical replicates (triplicate measurements) of this batch. The plant material was taxonomically identified, and a voucher specimen was deposited in the Prisco Bezerra Herbarium (EAC) under registration number EAC 67246.

The pods (Figure 1a) were manually opened, and the seeds (Figure 1b) were removed and selected based on physical integrity and apparent sanitary condition, with damaged or defective seeds discarded. After selection, seeds were cleaned by the dry removal of adhering dust and foreign material (manual sorting and brushing), without washing or chemical sanitization, to prevent moisture uptake prior to milling. The cleaned seeds were dried in a tray dryer at 50 °C for 4 h, cooled to room temperature, and then milled.

**Figure 1:**
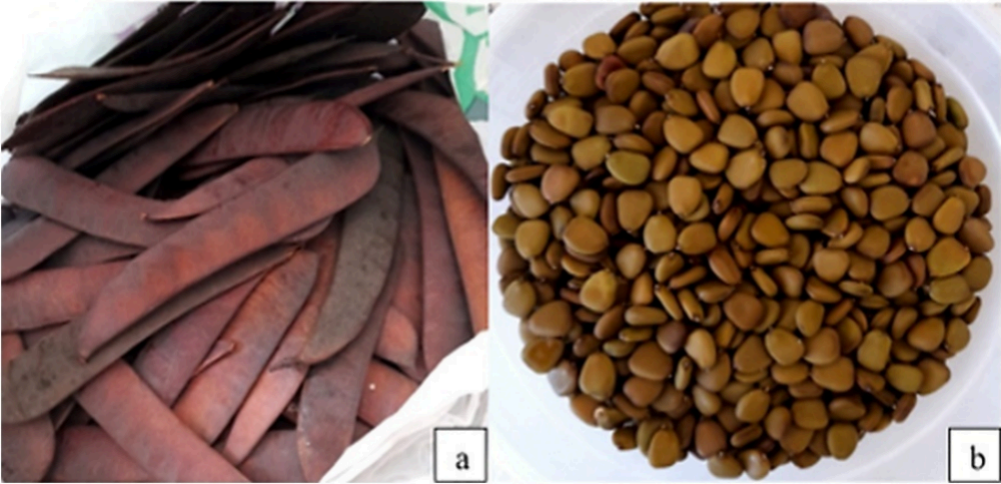
Pods (a) and seeds (b) of flamboyant-mirim (*Caesalpinia pulcherrima* (L.) Swartz) used to produce seed flour (FSF).

Seed coats were not removed prior to grinding. Milling was conducted at ambient temperature (28 ± 2 °C). Seeds were preground using an industrial blender (Model Li1.5, Skymsen, Brazil) in batches of 200 g per batch for a total of 5 min (1 min intervals) to minimize sample heating. The preground material was then milled in a Willey-type knife mill (CE-430, Cienlab, Brazil) equipped with 4 moving and 4 fixed knives, operated with an induction motor at a fixed rotation speed (∼1750 rpm) under room-temperature conditions.

The resulting flour was fractionated by dry sieving using a stainless-steel sieve stack (Bertel, Brazil) with mesh openings of 0.710, 0.500, 0.355, and 0.250 mm arranged from top to bottom (largest to smallest aperture), mounted on an electromagnetic sieve shaker (Rotachoc Chopin, France) and sieved for 10 min. Fractions corresponded to the material retained on each sieve: F0.710 (>0.710 mm; retained on 0.710 mm), F0.500 (0.500–0.710 mm; retained on 0.500 mm), F0.355 (0.355–0.500 mm; retained on 0.355 mm), and F0.250 (0.250–0.355 mm; retained on 0.250 mm). Material passing through 0.250 mm (pan fraction, <0.250 mm) was discarded. After granulometric separation, the flamboyant-mirim seed flour (FSF) fractions were vacuum-packed, protected from light, and stored at room temperature until further analyses.

### 2.2 Particle Size Analysis

The particle size distribution of FSF was determined according to the method described by the American Association of Cereal Chemists (AACC),^28^ with minor modifications. A 100 g portion of the sample was subjected to sieving using a set of standard sieves (Bertel, Brazil) with mesh openings of 0.710, 0.500, 0.355, and 0.250 mm placed over a collecting pan. The sieving procedure was carried out for 10 min using an electromagnetic sieve shaker (Rotachoc Chopin, France) operating at a constant vibration rate. The fraction retained on each sieve was expressed as the percentage of the initial sample mass retained on the respective sieve, calculated as

$$\mathrm{fraction}\;{\mathrm{retained}}_{i}\;(\%)=\left(\frac{m_{i}}{m_{0}}\right)\times 100$$ 1

where *m*_*i*_ is the mass retained on sieve *i* (g) and *m*_0_ is the initial sample mass subjected to sieving (g).

### 2.3 Physicochemical and Proximate Analyses

Physicochemical and proximate analyses of flamboyant-mirim seed flour (FSF) included pH, soluble solids, moisture, ash, lipids, proteins, and crude fiber, following official procedures from AOAC International.^29^ Briefly, pH was measured in an aqueous slurry (1:10 w/v, flour:distilled water) using a calibrated pH meter, and soluble solids were determined in the aqueous extract using a digital refractometer and expressed as °Brix. Moisture was determined by oven drying at 105 °C until constant mass (AOAC 925.10) and ash by incineration at 550 °C (AOAC 923.03). Total lipids were determined by Soxhlet extraction using hexane for 8 h (AOAC 920.39), and protein was determined by the Kjeldahl method (AOAC 979.09) using a nitrogen-to-protein conversion factor of 6.25. Crude fiber was determined by acid–alkali digestion according to AOAC 962.09. Water activity was measured using a water-activity analyzer (Nov-Labswift, Novasina, Switzerland) according to the manufacturer’s instructions. Carbohydrate content was calculated on an as-is basis (g per 100 g sample) by difference using the mean values of the measured components, according to

carbohydrates(g/100g)=100−(moisture+ash+protein+lipids+crudefiber) 2

Thus, representing nonfiber carbohydrates (excluding crude fiber). All proximate components were determined in triplicate, and carbohydrate values were computed from the mean component values.

The caloric value (kcal·100 g^–1^) was estimated using Atwater factors of 4 kcal g^–1^ for carbohydrates, 9 kcal g^–1^ for lipids, and 4 kcal g^–1^ for proteins.

Color parameters (L*, a*, and b*) were determined in triplicate using a Konica Minolta CR-400 colorimeter (Osaka, Japan) and expressed in the CIELAB color system, as recommended by the manufacturer. Apparent density was measured according to the method described by Kaur et al.,^30^ with results expressed as sample weight per unit volume (g·mL^–1^).

Unless otherwise stated, triplicates corresponded to analytical replicates (three repeated measurements) performed on the same homogenized flour fraction obtained from the composite batch.

### 2.4 Technological Properties

The water solubility index (WSI) of the flamboyant-mirim seed flour (FSF) was determined according to the method described by Du et al.^31^ Measurements were performed in triplicate, and the WSI was calculated as the weight of dissolved solids in the supernatant (g) divided by the sample weight (g) and multiplied by 100.

The water absorption capacity (WAC) was measured following the centrifugation method proposed by Kaur and Singh.^32^ WAC was calculated in triplicate as the weight of the sediment (g) divided by the initial sample weight (g).

Oil absorption capacity (OAC) was determined according to the method of Du et al.,^31^ also performed in triplicate, and expressed as the weight of absorbed oil (g) per gram of sample (g).

The emulsifying activity (EA) of the samples was determined following Kaur et al.^30^ Measurements were made in triplicate, and EA was calculated as the volume of the emulsion divided by the total volume multiplied by 100.

Foaming capacity (FC) was determined according to Bala et al.^27^ FC was evaluated in triplicate and calculated as the increase in foam volume, expressed as the difference between final and initial foam volumes, multiplied by 100 and divided by the initial foam volume.

### 2.5 Mineral Analysis

Mineral analyses were carried out according to the methods described by Silva and Queiroz.^33^ Approximately 200 mg (0.2 g) of each sample was weighed and transferred into digestion tubes, to which 5 mL of a nitroperchloric acid mixture (2:1, v/v) was added. The tubes were placed in a digestion block at 200 °C for approximately 2 h, until the extracts became clear and transparent, with a final volume of about 2 mL. Phosphorus (P) content was determined by homogenizing the digested extract in a vortex mixer and measuring absorbance at 725 nm by using a UV–visible spectrophotometer (BEL Photonics, Model 2000 UV, Brazil). Potassium (K) was quantified directly by using a flame photometer (Micronal, Model 906 AA, Brazil) after homogenization. For calcium (Ca), magnesium (Mg), iron (Fe), zinc (Zn), and manganese (Mn), the extracts were homogenized and analyzed by atomic absorption spectrophotometry (GBC, Model B462, Australia). The mineral concentrations were first expressed in parts per million (ppm) and then converted to g·kg^–1^ of sample (ppm = mg·kg^–1^).

Reagent blanks were included in all digestions (acid mixture without sample) to verify the background contributions. Instruments were calibrated using external multipoint standard curves prepared from certified single-element standard solutions covering the working concentration range for each analyte, and calibration linearity was verified prior to sample analysis. Calibration was periodically checked throughout the analytical sequence by using verification standards. Method performance was evaluated based on blank control, calibration verification, and replicate agreement. Limits of detection and quantification were estimated from blank variability (signal-to-noise approach) and used to confirm that all reported values were above the quantification level. A certified reference material was not analyzed in this study.

### 2.6 Determination of Total Phenolic Content and Antioxidant Capacity by the DPPH Method

Aqueous extracts from the different particle-size fractions of flamboyant-mirim seed flour (FSF) were prepared at a 1:10 (w/v) ratio (10 g of sample to 100 mL of solvent). Extractions were performed at ambient temperature (28 ± 2 °C). Samples were shaken on an orbital shaker (NovaTécnica, Model NT145, Brazil) at 90 rpm for 30 min, followed by sonication (LGI Scientific, Model LGI-LUC-180, Brazil) at 37 kHz for 30 min. To minimize heating during ultrasound treatment and avoid degradation of thermosensitive metabolites, the ultrasonic bath water was periodically renewed, consistent with common temperature-control practices reported for the ultrasound-assisted extraction of phenolics. After vacuum filtration through Whatman No. 4 filter paper (Prismatec, Model 132 type 2VC, Brazil), the filtrates were adjusted to the initial volume (100 mL) with distilled water to standardize the extract concentration across samples. Total phenolic content and antioxidant capacity were subsequently determined, and the results were normalized to sample mass.

Total phenolic content (TPC) was determined according to Swain and Hillis.^34^ Samples were dissolved in distilled water, and a 0.5 mL aliquot of each extract was transferred to a test tube containing 8 mL of distilled water and 0.5 mL of 20% (v/v) Folin–Ciocalteu reagent. After vortex mixing and a 3 min rest period, 1 mL of a 20% (m/v) sodium carbonate (Na_2_CO_3_) solution was added. The mixture was incubated in a water bath at 37 °C for 1 h, and absorbance was read at 720 nm using a spectrophotometer (Biosystems, Model SP2100/UV/5NM-IC, Spain). Gallic acid (Sigma, USA) was used as a standard to construct the calibration curve (*y* = 5.7467*x* + 0.014; *R* ^2^ = 0.9989). Total phenolic content was expressed as milligrams of gallic acid equivalents (GAE) per gram of sample. All measurements were performed in triplicate.

Antioxidant activity was determined by the DPPH• radical scavenging assay as described by Vieira et al.^35^ and Brand-Williams et al.^36^ A reaction mixture containing 1.5 mL of an ethanolic DPPH• solution (6 × 10^–5^ M) and 0.5 mL of each extract was prepared. For EC_5_0 determination, each extract was tested using a serial dilution (1:2, v/v) to obtain six to eight concentration levels (0.25–8.00 mg mL^–1^, depending on the extract activity), ensuring that the inhibition responses encompassed values below and above 50%. Concentrations are reported as the final extract concentration in the reaction mixture. After 30 min of reaction in the dark, the absorbance was measured at 517 nm using the same spectrophotometer. All analyses were carried out in triplicate, including a negative control (without antioxidant) and two positive controls (ascorbic acid and Trolox, representing natural and synthetic antioxidants, respectively). The percentage of DPPH• inhibition was calculated relative to that of the control. EC_50_ (effective concentration required to inhibit 50% of DPPH•) was estimated by nonlinear regression of the inhibition (%) versus concentration curve using a four-parameter logistic (4PL) model, and goodness-of-fit was evaluated by *R* ^2^ and residual inspection.

### 2.7 Nuclear Magnetic Resonance (NMR) Analysis

For quantitative analysis using proton nuclear magnetic resonance (^1^H NMR), 20 mg of flamboyant-mirim seed flour with a particle size of 0.250 mm was mixed directly with 600 μL of the following deuterated solvents: chloroform-d (99.8%, Sigma-Aldrich), methanol-d_4_ (99.8%, Sigma-Aldrich), and deuterium oxide (D_2_O, 99.9%, Merck, Darmstadt, Germany). The mixtures were briefly sonicated for 1 min and then centrifuged for 1 min. The resulting supernatants were transferred to 5 mm NMR tubes.

The NMR experiments were performed on an Agilent 600 MHz spectrometer equipped with a 5 mm One Probe inverse detection probe (^1^H–^19^F/^15^N–^31^P) under quantitative analytical conditions: spectral acquisition was performed in triplicate with 32 scans at a controlled temperature to 298 K, and using the PRESAT pulse sequence for nondeuterated water suppression (4.79 ppm); hard pulse (P_1_) calibrated to 90° (8.75 μs pulse length at 58 dB power); acquisition time (AQ) of 5 s and relaxation delay (d_1_) of 20 s determined using the inversion–recovery pulse sequence ensuring 99.9% nuclear relaxation (7 T_1_).^37^ The TMSP-*d*_4_ was used as an internal standard (δ 0.0). Free induction decays were multiplied by an exponential function equivalent to 0.3 Hz line-broadening before applying a Fourier transform for 32,000 points, concerning the accepted error, signal resolution, and S/N amplification. Phase correction was manually performed, and the baseline correction was applied over the entire spectral range.

The identification of organic compounds in the chloroformic, methanolic, and aqueous extracts was achieved through two-dimensional NMR experiments (^1^H–^1^H gCOSY, ^1^H–^13^C gHSQC, and ^1^H–^13^C gHMBC). Compound assignments were made using data from open-access databases (www.hmdb.ca) and previously published literature.^16,38^ Compounds with nonoverlapping signals in the ^1^H NMR spectra were quantified.

### 2.8 Statistical Analysis

#### 2.8.1 Univariate Statistical Analysis

All experiments related to physicochemical, proximate, technological, mineral, total phenolic, antioxidant, and ^1^H NMR quantification analyses were performed in triplicate. Results were expressed as mean ± standard deviation (SD). One-way analysis of variance (ANOVA) and Tukey’s multiple comparison test were applied at a 5% significance level (*p* < 0.05) to evaluate statistical differences among the samples for each analytical parameter. These analyses were conducted using MINITAB statistical software, version 19.0 (Minitab Inc., State College, PA, USA).

#### 2.8.2 Multivariate Statistical Analysis

Supervised multivariate statistical analysis was performed using partial least squares-discriminant analysis (PLS-DA), considering flour particle sizes as classification groups. The data set included physicochemical, proximate, technological, mineral, and functional parameters. The data matrix was imported into the PLS-Toolbox software (version 8.6.2, eigenvector Research Inc., Manson, WA, USA). Prior to model construction, the data were autoscaled (mean-centered and variance-scaled). The Simplified PLS (SIMPLS) algorithm was applied to decompose the complex data matrix into scores, loadings, and model performance parameters. Relevant information was obtained using four latent variables (4 LVs), with cross-validation performed at a 95% confidence level using the Venetian Blinds method. The optimal number of LVs was determined based on total variance captured, root-mean-square error of calibration (RMSEC) and cross-validation (RMSECV), and bias values obtained on modeling calibration and validation.

## 3 Results and Discussion

The particle size distribution of flamboyant-mirim seed flour (FSF) showed a nonuniform pattern, with significant differences among the granulometric fractions (*p* < 0.05), indicating that the milling and sieving procedures effectively produced distinct particle-size classes. The lowest proportion of retained material (17.64%) was associated with particles around 0.500 mm, whereas the highest retention was observed in the intermediate fractions, with 28.43% and 29.37% corresponding to particles of approximately 0.710 mm and 0.355 mm, respectively.

Regarding the yield, the finest fraction (0.250 mm) represented approximately 25% of the total flour mass obtained after milling and sieving. Although it is not the major mass fraction, its yield is practically relevant, because it corresponds to a substantial portion of the material generated by a simple dry fractionation step. Importantly, this fraction concentrated several desirable attributes (higher ash, protein, lipids, fiber, and mineral contents, as well as improved technological properties such as water solubility index, oil absorption capacity, foaming, and emulsifying performance). From an application standpoint, this indicates that particle-size classification can be used as a straightforward strategy to obtain a nutrient- and functionality-enriched ingredient stream without additional chemical processing, while the remaining fractions may still be directed to other formulations depending on the targeted technological and bioactive profile.

This pattern suggests a heterogeneous milling behavior of *Caesalpinia pulcherrima* seeds, possibly related to the composition and structural rigidity of the cotyledons and seed coat. Coarser fractions (0.710 mm) are often associated with the retention of particles that better preserve the inherent seed matrix and tend to show higher dietary-fiber contribution, whereas intermediate fractions (0.355 mm) may contain a higher proportion of more uniform starchy/proteinaceous material, depending on the botanical structure and milling conditions.^39,40^ The lower yield in the 0.500 mm fraction may be due to the transitional nature of this size range, where particles are either too large to pass through smaller meshes or too fine to remain on larger meshes, resulting in less material accumulation.

From a technological standpoint, particle size distribution plays a decisive role in determining the flour functionality. Finer flours typically show higher specific surface area, improving hydration, solubility, and reactivity with other food components, which may influence subsequent parameters such as water absorption capacity, emulsifying behavior, and color uniformity.^41^ Coarser fractions, on the other hand, can enhance texture and fiber content in bakery and extruded products.^42^ Therefore, understanding the granulometric profile of FSF provides a fundamental basis for predicting its functional performance and optimizing its incorporation into food formulations.

### 3.1 Physicochemical and Proximate Composition

The physicochemical and proximate composition results of flamboyant-mirim seed flour (FSF) are presented in Table 1. It can be observed that particle size significantly influenced the physicochemical and proximate properties of FSF (*p* < 0.05). Variations among the granulometric fractions indicate that milling and sieving affected the distribution of nutritional and structural components within the flour matrix.

**Table 1:** ANOVA Evaluation Considering the Physicochemical and Proximate Composition of Flamboyant-Mirim (*Caesalpinia pulcherrima* (L.) Swartz) Seed Flour (FSF) at Different Particle Sizes^a^

	**particle size (mm)**			
**parameters**	0.710	0.500	0.355	0.250
**physicochemical**				
pH	6.10 ± 0.06^b^	5.85 ± 0.08^c^	6.24 ± 0.08^ab^	6.29 ± 0.07^a^
water activity (aw)	0.49 ± 0.00^a^	0.49 ± 0.00^a^	0.49 ± 0.00^a^	0.49 ± 0.01^a^
soluble solids (°Brix)	0.50 ± 0.1^b^	1.46 ± 0.15^a^	1.40 ± 0.10^a^	1.63 ± 0.05^a^
L*	68.32 ± 0.07^c^	67.15 ± 0.28^d^	69.17 ± 0.21^b^	71.15 ± 0.08^a^
a*	2.51 ± 0.03^a^	2.31 ± 0.07^bc^	2.26 ± 0.05^c^	2.40 ± 0.03^ab^
b*	15.30 ± 0.31^c^	15.00 ± 0.32^c^	16.72 ± 0.15^b^	18.01 ± 0.09^a^
bulk density (g·mL^–1^)	0.77 ± 0.00^a^	0.69 ± 0.02^b^	0.65 ± 0.02^bc^	0.61 ± 0.02^c^
**proximate composition**				
ash (%)	1.99 ± 0.03^d^	2.91 ± 0.04^c^	3.57 ± 0.03^b^	3.85 ± 0.00^a^
moisture (%)	7.41 ± 0.83^a^	6.52 ± 0.47^a^	6.11 ± 0.77^a^	5.92 ± 0.12^a^
lipids (%)	3.75 ± 0.61^c^	6.35 ± 0.70^b^	8.69 ± 0.80^a^	9.90 ± 0.40^a^
protein (%)	9.35 ± 0.17^d^	17.44 ± 0.17^c^	23.80 ± 0.11^b^	27.34 ± 0.78^a^
fibers (%)	0.55 ± 0.17^c^	0.88 ± 0.16^bc^	1.10 ± 0.07^ab^	1.32 ± 0.11^a^
carbohydrates (%)	76.93 ± 1.29^a^	65.89 ± 0.66^b^	56.71 ± 0.87^c^	51.66 ± 1.27^d^
caloric value (kcal·100 g^–1^)	379.00 ± 4.74^c^	390.53 ± 5.33^bc^	400.34 ± 6.64^ab^	405.10 ± 1.35^a^

a Different letters in the same row indicate statistically significant differences according to Tukey’s test (*p* < 0.05).

Finer fractions often exhibit higher concentrations of ash, proteins, and lipids, which may be attributed to the greater exposure of internal cellular constituents and the enrichment of denser particles after sieving.^43,44^ Conversely, coarser fractions may retain higher amounts of carbohydrates (starch-rich particles) and, depending on milling and dehulling conditions, fiber-rich fragments associated with the seed coat/pericarp, reflecting the predominance of cell wall material in larger particles.^43,45^ Such behavior is consistent with the heterogeneous composition of legume seeds, in which macro- and micronutrients are unevenly distributed between cotyledon and seed coat layers.^46^

The influence of particle size on physicochemical parameters such as pH, water activity, and soluble solids suggests possible structural and compositional rearrangements induced by the milling process. Reduced particle size enhances the surface area, potentially increasing the interaction of hydrophilic components with water and modifying the hydration properties of the flour.^47^ These effects are of technological relevance, as they may impact the functional behavior of FSF when applied as an ingredient in food systems, especially those involving hydration, gelation, or emulsification mechanisms.

A decreasing pH with smaller particle sizes has been reported for pea flours, which has been attributed to compositional redistribution during milling/sieving, including a higher exposure of intracellular constituents and buffering compounds in finer streams, as well as differences in the relative contribution of seed coat fragments across fractions.^19^ In the present study, however, pH did not follow a monotonic trend across particle sizes, suggesting that the balance between buffering components (e.g., proteins/minerals) and acidic constituents may vary among fractions and depends on both the seed microstructure and milling conditions. In contrast, studies by Savlak et al.^48^ and Nabil et al.^49^ found that particle size distribution did not significantly influence the pH values of green banana and cladode flours, respectively.

The water activity (aw) values of flamboyant-mirim seed flours (FSF) ranged from 0.490 to 0.496, indicating a microbiologically stable product. These low values (<0.60) limit the growth and multiplication of microorganisms.^50^ The granulometric fractions of FSF were not significantly affected (*p* > 0.05) by particle size. Similarly, Ahmed et al.^51^ reported that particle size reduction did not significantly affect the water activity of rice flours, suggesting that, under low-moisture conditions, aw is primarily governed by the overall moisture content and the water-binding capacity of the matrix rather than by particle size alone. In contrast, particle size was reported to influence aw in green banana flour^48^ and okra seed flour,^52^ which has been associated with matrix-dependent factors such as differences in hygroscopic components (soluble carbohydrates and fiber), surface area and porosity, and the relative proportion of cellular wall fragments exposed after milling. Therefore, the effect of particle size on aw is not universal and depends on the composition and microstructure of each flour.^53^ In the present study, the lack of significant differences in aw among FSF fractions indicates that the granulometric separation did not substantially alter the balance between free and bound water under the conditions evaluated.

The soluble solids (SS) content of flamboyant-mirim seed flour (FSF) fractions ranged from 0.50 to 1.63 °Brix, and particle size had a statistically significant effect (*p* < 0.05). The 0.500 mm and 0.250 mm fractions exhibited higher SS values compared with the 0.710 mm fraction. This difference may be associated with milling-induced starch damage and partial depolymerization, which increase starch solubility and favor the formation/release of low-molecular-weight, water-soluble carbohydrates (dextrins and sugars) that can be more prevalent in finer flour streams.^54–56^ In addition to sugars, soluble solids are also influenced by the presence of organic acids.^57^

Similar variations in soluble solids content with decreasing particle size have been reported for cladode flour^49^ and green banana flour,^48^ supporting the influence of granulometric reduction on the concentration of soluble components.

The color parameter results for flamboyant-mirim seed flour (FSF) fractions are shown in Table 1. The lightness (L*) values ranged from 67.15 to 71.15, indicating a tendency toward lighter or whitish coloration. The finest fraction (0.250 mm) exhibited the highest L* value among all samples. This increase in lightness for smaller particle sizes may be attributed to the greater surface area, which enhances light reflection.^36^ Similar behavior has been reported for green banana flour.^48^

The redness index (a*) of FSF varied between 2.26 and 2.51, with a general tendency toward a reddish hue. A decrease in a* values was observed as the particle size decreased. This reduction may be related to the degradation or dilution of pigments during the milling process.^52^ In contrast, Jiang et al.^58^ reported that particle size had no significant effect on the a* parameter of *Vaccinium bracteatum* Thunb. leaf flour. For the yellowness parameter (b*), values ranged from 15.00 to 18.01, indicating a predominance of yellow tones. The 0.250 mm fraction showed the highest b* value (18.01), consistent with observations in pea flour.^27^ The increase in the b* intensity may be associated with pigment oxidation processes involving phenolic compounds, ascorbic acid, and carotenoids, which contribute to the yellowish coloration of the flour.

Bulk density determines the expansion and packing behavior of the flours. The apparent density of flamboyant-mirim seed flour (FSF) ranged from 0.61 to 0.77 g·mL^–1^. A decrease in density was observed as the particle size decreased. Similar trends were reported for pea flour^27^ and *Vaccinium bracteatum* Thunb. leaf flour.^58^ High bulk density values suggest that FSF is suitable for use in dense food preparations, whereas low bulk density can be advantageous in the formulation of complementary or infant foods, where lightness and dispersibility are desirable.^59^

Ash content in FSF fractions ranged from 1.99% to 3.85% (Table 1). For *C. pulcherrima*, similar ash levels have been reported for seed flours/meals prepared from whole seeds or dehulled kernels,^60,41^ whereas higher values were observed in studies analyzing whole-seed meals or processed seed flours from the same species.^8,9,61^ Differences among literature values are expected because ash content is strongly influenced by seed portion (whole seed vs dehulled kernel), processing conditions, and analytical basis. In our samples, ash increased with decreasing particle size, suggesting preferential enrichment of mineral-rich particles in finer fractions during milling and sieving, as also reported for size-fractionated quinoa flour, in which finer fractions exhibited higher ash content.^62^ In contrast, particle size reduction did not significantly affect ash content in pea flour^27^ or rice flour,^63^ indicating that the impact of size classification on ash can be matrix-dependent.

Moisture values ranged from 5.92% to 7.41%, with no significant differences (*p* > 0.05) among FSF fractions. Higher moisture contents were reported by Chiodetti et al.,^64^ while lower values were observed by Sahu et al.^60^ The moisture levels obtained in all FSF fractions comply with the limits established by the Brazilian regulatory standard RDC no. 711,^65^ which sets a maximum moisture content of 15% for flours, starches, and cereal brans.

The lipid content of FSF fractions ranged from 3.75% to 9.90% (Table 1). When comparing with the literature, it is important to note that reported lipid levels for *C. pulcherrima* often refer to different sample types, such as whole-seed meals, dehulled kernel (“seed nut”) flours, or processed seed flours, which can affect the measured lipid fraction.^8,16,61^ In our material, lipid content increased with decreasing particle size, which is consistent with the compositional partitioning that occurs during milling/sieving: finer fractions tend to be enriched in cotyledon-derived particles and intracellular constituents, and the reduction in particle size increases cell disruption and the extractability of lipids by enhancing the surface area and solvent access. This behavior has also been reported for other milled/fractionated matrices, such as β-d-glucan concentrates from barley and rice flours/cultivars, where finer fractions displayed higher lipid contents, reflecting both enrichment of lipid-containing particles and improved extraction efficiency.^66^

Protein content ranged from 9.35% to 27.34%. Comparable results were reported by Omode et al.,^61^ while higher levels were found by Oderinde et al.^16^ and Yusuf et al.^8^ The 0.250 mm fraction exhibited the highest protein content (27.34%). This enrichment in the finer fraction may indicate that the protein-rich parts of the seed were broken into smaller particles during milling.^66^ Similar findings were observed for β-d-glucan concentrates from barley and quinoa flour.^62^ However, Ahmed et al.^51^ reported a decrease in protein content with smaller particle sizes in rice flour.

Crude fiber contents ranged from 0.55% to 1.32%, which were lower than those reported by Oderinde et al.^16^ and Yusuf et al.^8^ An increase in fiber content was observed with decreasing particle size, which may be associated with the cleavage of intermolecular bonds, disruption of protein structures, and solubilization of macromolecules during milling.^67^ Similar increases in fiber content with particle size reduction have been documented for β-d-glucan concentrates from barley and quinoa flour.^51,62^

Carbohydrate content varied from 51.66% to 76.93%, higher than the values reported by Oderinde et al.,^16^ Yusuf et al.,^8^ and Omode et al.^61^ A decrease in the carbohydrate content was observed with decreasing particle size. Since carbohydrate values were obtained by differences, this variation is closely related to the distribution of moisture, protein, lipid, ash, and fiber contents among FSF fractions (Table 1), all of which are affected by milling and sieving. In contrast to the present findings, Memon et al.^68^ reported an increase in carbohydrate content with smaller particle size in whole wheat flour, while Bala et al.^27^ observed no significant effect in pea flour.

The caloric values of FSF fractions ranged from 379.00 to 405.10 kcal·100 g^–1^. Similar results were reported by Oderinde et al.^16^ An increase in the caloric value was observed with decreasing particle size, which may be attributed to the higher concentrations of proteins, lipids, and carbohydrates in finer fractions. A similar trend was observed in wheat flour.^68^ However, Bala et al.^27^ reported no significant differences in caloric values with particle size reduction in pea flour.

### 3.2 Technological Properties

The results of the technological properties are presented in Table 2. The water solubility index (WSI) of FSF fractions ranged from 2.14 to 11.65 g·100 g^–1^. An increase in WSI was observed as the particle size decreased. This variation in solubility index may be attributed to the presence of dispersible molecules such as albumins, amylose, sugars, oligosaccharides, and other soluble constituents.^38^ Similarly, an increase in WSI values with decreasing particle size has been reported for pea flour.^27^ In contrast, a reduction in WSI values was observed for cladode flour,^26^ suggesting that the effect of particle size on solubility may depend on the intrinsic composition and structural characteristics of each raw material.

**Table 2:** ANOVA Evaluation Considering the Technological Properties of Flamboyant-Mirim (*Caesalpinia pulcherrima* (L.) Swartz) Seed Flour (FSF) at Different Particle Sizes^a^

	**particle size (mm)**			
**parameters**	0.710	0.500	0.355	0.250
water solubility index (WSI) (g·100 g^–1^)	2.14 ± 0.70^d^	4.45 ± 0.32^c^	6.58 ± 0.78^b^	11.65 ± 0.52^a^
water absorption capacity (WAC) (g·g^–1^)	5.08 ± 0.19^a^	4.14 ± 0.28^b^	3.60 ± 0.13^b^	2.94 ± 0.21^c^
oil absorption capacity (OAC) (g·g^–1^)	1.48 ± 0.04^b^	1.60 ± 0.03^ab^	1.58 ± 0.07^ab^	1.71 ± 0.03^a^
emulsifying activity (%)	57.14 ± 0.00^d^	64.28 ± 0.00^c^	67.85 ± 0.00^b^	80.95 ± 2.06^a^
foaming capacity (%)	2.00 ± 0.00^c^	2.00 ± 0.00^c^	10.00 ± 0.00^b^	14.66 ± 1.68^a^

a Different letters in the same row indicate statistically significant differences according to Tukey’s test (*p* < 0.05).

The WAC of FSF fractions ranged from 2.94 to 5.08 g·g^–1^. A decrease in WAC values was observed with a decreasing particle size. Similarly, a reduction in WAC with smaller particle size has been reported for green banana flour^48^ and cladode flour.^49^ The lower water absorption capacity in finer fractions may be attributed to the reduced availability of polar amino acids, whereas higher WAC values are often associated with greater amylose leaching, starch solubility, and the loss of crystalline structure.^59^

The OAC of FSF fractions ranged from 1.48 to 1.71 g·g^–1^. An increase in the level of OAC was observed with decreasing particle size, with the coarser 0.710 mm fraction showing the lowest value and the finer 0.250 mm fraction exhibiting the highest. Similar findings were reported for pea flour.^27^ The higher OAC in finer fractions may be related to the greater presence of hydrophobic proteins and lipid compounds, which enhance oil retention.^31,67^ This trend is consistent with the proximate composition results (Table 1), where the 0.250 mm fraction showed the highest protein and lipid contents.

The emulsifying activity (EA) of FSF fractions ranged from 57.14% to 80.95%. EA increased with decreasing particle size, which can be attributed to the higher specific surface area and improved dispersibility of smaller particles, facilitating faster adsorption of surface-active components at the oil–water interface and promoting the formation of a more cohesive interfacial film.^69,70^

The foaming capacity (FC) of FSF fractions ranged from 2.00% to 14.66%, with an increase in FC observed as the particle size decreased. Conversely, Bala et al.^27^ found a reduction in FC with smaller particle sizes in pea flour. The higher FC value observed for the 0.250 mm fraction may be associated with the greater concentration of foaming agents, mainly proteins, present in finer flours (Table 1). Since foaming properties depend largely on protein content and its ability to form stable films at the air–water interface, this characteristic highlights the functional potential of the finer FSF fractions.^27,71^

### 3.3 Mineral Composition

The mineral composition of the different particle size fractions of FSF is presented in Table 3. It can be observed that particle size significantly influenced the mineral content of FSF (*p* < 0.05).

**Table 3:** ANOVA Evaluation Considering the Mineral Composition of Flamboyant-Mirim (*Caesalpinia pulcherrima* (L.) Swartz) Seed Flour (FSF) at Different Particle Sizes^a^

	**particle size (mm)**			
minerals (g·kg^**–1**^ **)**	0.710	0.500	0.355	0.250
Ca	1.61 ± 0.00^d^	2.00 ± 0.00^c^	2.15 ± 0.00^b^	2.70 ± 0.00^a^
Mg	1.19 ± 0.01^d^	2.01 ± 0.19^c^	2.32 ± 0.00^b^	2.62 ± 0.02^a^
K	6.22 ± 0.22^c^	9.96 ± 0.21^b^	12.10 ± 0.13^a^	12.31 ± 0.13^a^
P	1.74 ± 0.01^d^	3.40 ± 0.03^c^	4.84 ± 0.17^b^	5.59 ± 0.02^a^
Fe	0.03 ± 0.00^d^	0.05 ± 0.00^c^	0.06 ± 0.00^b^	0.07 ± 0.00^a^
Mn	0.01 ± 0.00^d^	0.01 ± 0.00^c^	0.01 ± 0.00^b^	0.01 ± 0.00^a^
Zn	0.02 ± 0.00^d^	0.03 ± 0.00^c^	0.04 ± 0.00^b^	0.05 ± 0.00^a^

a Different letters in the same row indicate statistically significant differences according to Tukey’s test (*p* < 0.05).

Calcium (Ca) content ranged from 1.61 to 2.70 g/kg across the granulometric fractions, while magnesium (Mg) varied from 1.19 to 2.62 g/kg. When compared with previous data for *C. pulcherrima*, it is important to note that the literature often reports mineral values for whole seeds or seed nuts (dehulled kernels) rather than for sieved flour fractions. For example, Yusuf et al. evaluated *C. pulcherrima* whole seeds and seed nuts, reporting the relative abundance of minerals and differences between these seed portions, which makes direct comparison with fractionated flours nontrivial.^8^ Likewise, Agbede^22^ investigated processed *C. pulcherrima* seed flour obtained after dehulling and thermal processing, showing that processing can modify the proximate profile and mineral levels.

In FSF, K was the most abundant macromineral (6.22–12.31 g/kg), followed by P (1.74–5.59 g/kg), and the overall increase in Ca, Mg, K, P, Fe, Mn, and Zn with decreasing particle size indicates mineral enrichment in finer fractions. This pattern is consistent with reports on dry fractionation/size classification of pulse flours, in which fine fractions become enriched not only in protein and lipids but also in minerals/ash due to preferential partitioning of smaller, denser, nutrient-rich particles during milling and classification.^72,73^ Therefore, the higher mineral concentrations observed in finer FSF fractions are consistent with compositional partitioning during milling and sieving, rather than representing a simple “size effect” alone.

From a structural standpoint, such a redistribution is expected because legume seeds are heterogeneous tissues: the cotyledon and the seed coat differ markedly in composition, and seed coats are typically rich in dietary fiber and may carry substantial amounts of minerals and phytochemicals. Therefore, the extent to which seed coat fragments and associated cellular materials is incorporated into each size fraction can influence mineral profiles.^7,74^ In addition, mineral enrichment in fine fractions often correlates with higher protein levels, as protein-rich particles may coseparate with minerals during milling/fractionation; similar associations between protein and mineral contents have been reported in milled cereal flours and refined fractions^75^ and are broadly discussed for pulse fractionation processes.^43^

The FSF fractions demonstrated appreciable levels of several essential minerals, particularly in the 0.250 mm fraction, which exhibited the highest concentrations. These findings highlight the nutritional potential of flamboyant-mirim seed flour as a promising ingredient for fortifying food formulations with macro- and microminerals of dietary relevance.

### 3.4 Phenolic Content and Antioxidant Activity

The results of total phenolic content and in vitro antioxidant capacity analyses of the aqueous extracts from the different particle size fractions of flamboyant-mirim seed flour (FSF) are presented in Table 4. It can be observed that particle size significantly (*p* < 0.05) influenced both total phenolic content and antioxidant activity of the FSF fractions.

**Table 4:** ANOVA Evaluation Considering the Total Phenolic Content and In Vitro Antioxidant Capacity (EC_50_ in mg·mL^–1^) of Aqueous Extracts from Different Particle Size Fractions of FSF^a^

**aqueous extracts from the particle size fractions of FSF**	total phenolics (mg GAE·100 g^**–1**^ **)**	antioxidant capacity (EC_50_ mg·mL^**–1**^ **)**
0.710 mm	28.28 ± 0.54^d^	0.67 ± 0.01^a^
0.500 mm	53.35 ± 0.76^a^	0.18 ± 0.00^d^
0.355 mm	34.14 ± 0.25^c^	0.43 ± 0.01^c^
0.250 mm	39.00 ± 0.87^b^	0.58 ± 0.00^b^

a Different letters in the same row indicate statistically significant differences according to Tukey’s test (*p* < 0.05).

The total phenolic content of the aqueous extracts from the different particle size fractions of flamboyant-mirim seed flour (FSF) ranged from 28.28 to 53.35 mg of GAE·100 g^–1^ (Table 4). Lower values were reported by Dela Torre,^76^ while higher concentrations were observed by Chanda et al.^9^ and Sahu et al.^60^

Regarding particle size, the 0.500 mm fraction exhibited the highest total phenolic content, whereas the 0.710 mm fraction showed the lowest value (*p* < 0.05). The reduced phenolic content in the coarser fraction may be associated with its higher carbohydrate concentration (Table 1), as suggested by Becker et al.,^77^ who reported that larger particle fractions tend to contain more carbohydrates and consequently fewer bioactive compounds.

The antioxidant capacity (EC_50_) of the aqueous FSF extracts ranged from 0.18 to 0.67 mg·mL^–1^ (Table 4). Similar results were reported by Chanda et al.^9^ and Dela Torre et al.^76^ Since lower EC_50_ values indicate higher antioxidant potential,^77^ the 0.500 mm fraction displayed the greatest antioxidant activity (0.18 mg·mL^–1^), whereas the 0.710 mm fraction exhibited the lowest (0.67 mg·mL^–1^). Variations in antioxidant activity may be attributed to differences in the underlying reaction mechanisms of the assays used, as well as to the varying reactivity of individual components within the extracts.^78^

No consistent trend was observed between the particle size and the total phenolic or antioxidant capacity values of FSF fractions. Similar findings were reported for green banana flour of different particle sizes, in which no clear relationship was established between particle size and antioxidant activity.^48^ However, other researchers have suggested that particle size can influence the availability and extractability of phenolic compounds.^52,68,79,80^

The antioxidant activity of legumes is directly related to their total phenolic content.^81^ Accordingly, phenolic compounds contributed significantly to the antioxidant activity observed in the FSF particle size fractions. This finding is consistent with the general understanding that the antioxidant activity of plant-derived products is largely attributed to the radical-scavenging ability of phenolic compounds such as flavonoids, polyphenols, and tannins.^82^

### 3.5 NMR Analysis

Due to the superior proximate composition of the 0.250 mm FSF fraction and the overall similarity of the extract profiles across particle sizes observed in preliminary screening, this fraction was selected for deeper molecular-level characterization by nuclear magnetic resonance (NMR) spectroscopy. Figure 2 compares the ^1^H NMR spectra of extracts prepared by using different deuterated solvents: chloroform (a), methanol (b), and water (c). The compound-characterization parameters and assignments are summarized in Table 5. In addition, Figure 2a shows the chemical structure of a representative triacylglycerol containing both unsaturated and saturated fatty acids, which were identified as the major lipid-related constituents, particularly in the chloroformic (and to a lesser extent methanolic) extracts.

**Table 5:**
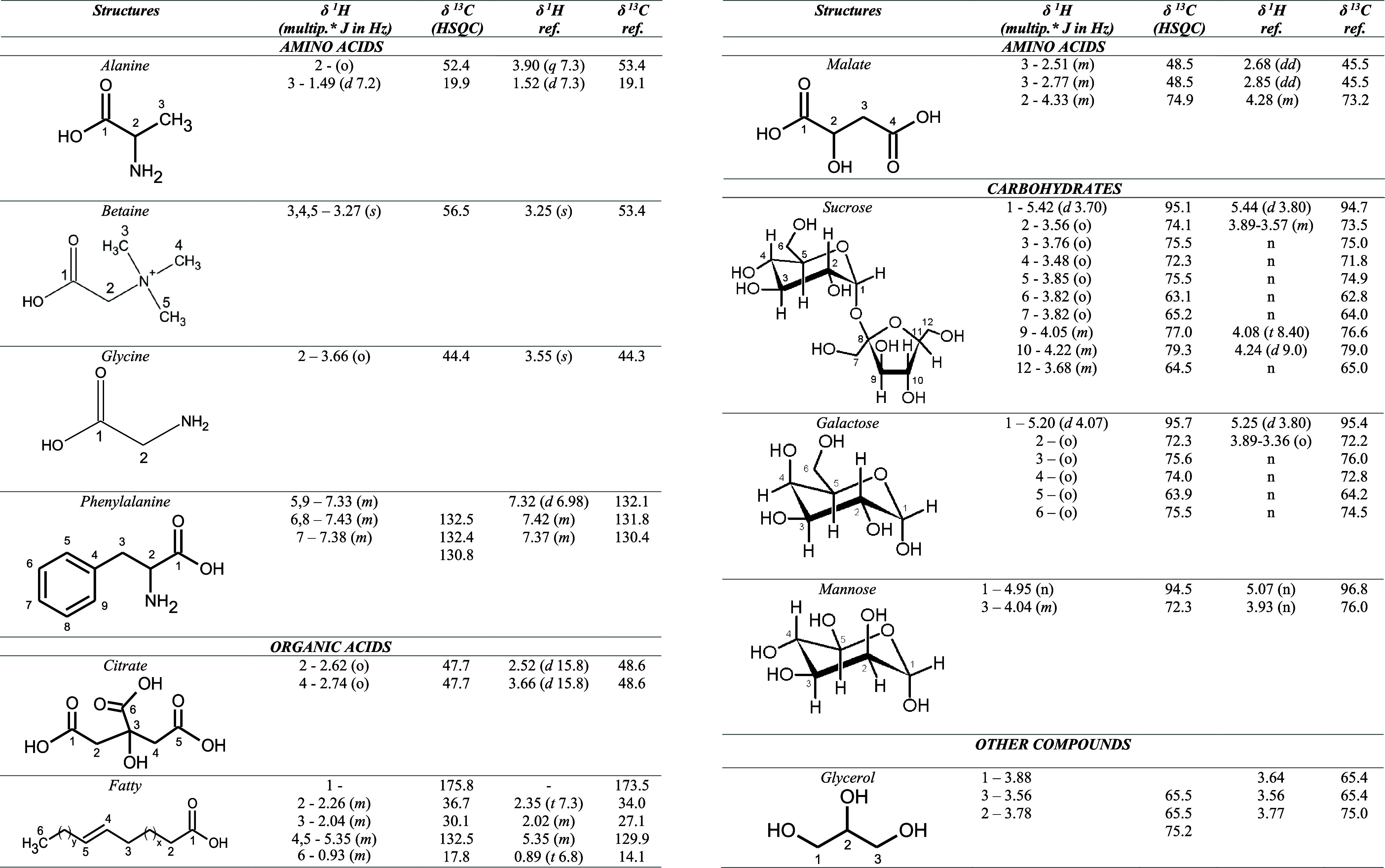
NMR Parameters of the Identification of the Organic Compounds in Extracts from Flamboyant-Mirim Seed Flour (0.250 mm) Obtained Using Deuterated Chloroform, Methanol, and Water: Chemical Structure, Experimental and Reference ^1^H and ^13^C Chemical Shifts, Signal Multiplicity, and Constant Coupling^a^

a *s* – simplet; *d* – duplet; *t* – triplet; *q* – quadruplet; *quin* – quintet; *dd* – double of duplets; *dt* – double of triplets; o – overlapping signal; n – no information; no – not observed.

**Figure 2:**
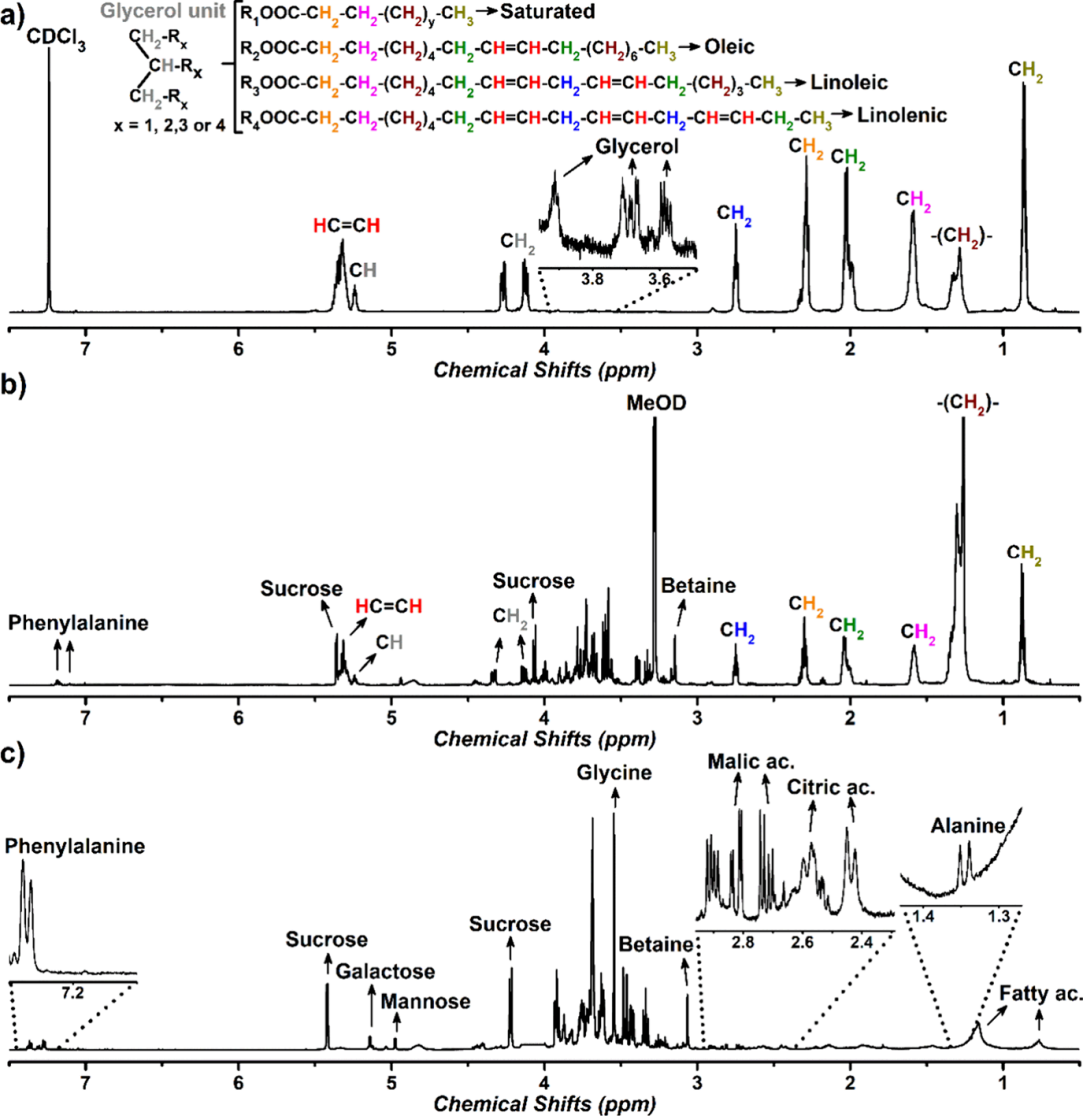
Comparison of ^1^H NMR spectra of flamboyant-mirim (*Caesalpinia pulcherrima* (L.) Swartz) seed flour (0.250 mm) obtained using different deuterated extraction solvents: chloroform (a), methanol (b), and water (c).

After a general understanding of the compositional variability among the flamboyant-mirim seed extracts was obtained, quantitative ^1^H NMR (^1^H *q*NMR) analysis was performed to complement the qualitative findings. Table 5 presents the concentrations of phenylalanine, unsaturated fatty acids, sucrose, galactose, mannose, the glycerol unit in the triacylglycerol structure, betaine, cysteine, citric acid, and alanine in the extracts. Values with different superscript letters in the same row indicate statistically significant differences (*p* < 0.05) according to one-way ANOVA, whereas values followed by the same letter are not significantly different.

As shown in Table 6, among the analyzed extracts, the aqueous extract exhibited significantly (*p* < 0.05) higher concentrations of most identified organic compounds compared with the chloroformic and methanolic extracts. Sucrose was identified as the predominant compound class, whereas unsaturated fatty acids and glycerol units were detected in higher proportions in the chloroformic extract.

**Table 6:** ANOVA Evaluation Considering the Concentrations of Organic Compounds Identified by Quantitative ^1^H NMR (^1^H *q*NMR) in mg per 100 mg of Chloroformic, Methanolic, and Aqueous Extracts of Flamboyant-Mirim (*Caesalpinia pulcherrima* (L.) Swartz) Seed Flour with a Particle Size of 0.250 mm^a^

	**FSF extract**		
identified compounds (mg·100 mg^**–1**^ **)**	**chloroformic**	**methanolic**	**aqueous**
unsaturated fatty acids	12.041 ± 0.138^a^	2.905 ± 0,022^b^	n.d.
glycerol unit	2.438 ± 0.031^a^	0.605 ± 0.007^b^	n.d.
phenylalanine	n.d.	0.157 ± 0,002^a^	0.664 ± 0.008^b^
betaine	n.d.	0.172 ± 0.002^a^	0.441 ± 0.005^b^
cysteine	n.d.	0.228 ± 0.002^a^	1.361 ± 0.014^b^
alanine	n.d.	n.d.	0.216 ± 0.002
sucrose	n.d.	4.227 ± 0.032^a^	20.095 ± 0.19^b^
galactose	n.d.	n.d.	2.646 ± 0.030
mannose	n.d.	n.d.	1.953 ± 0.017
citric acid	n.d.	n.d.	1.729 ± 0.018

a n.d.: not detected. Different letters in the same row indicate statistically significant differences according to Tukey’s test (*p* < 0.05).

Regarding unsaturated fatty acids, the FSF showed the highest concentration in the chloroformic extract (12.041 mg·100 mg^–1^) and the lowest in the methanolic extract (2.905 mg·100 mg^–1^). The glycerol unit within the triacylglycerol structure was also more abundant in the chloroformic extract (2.438 mg·100 mg^–1^) compared to the methanolic one (0.605 mg·100 mg^–1^).

Phenylalanine was identified in both methanolic and aqueous extracts of FSF, with concentrations ranging from 0.157 to 0.664 mg·100 mg^–1^, respectively. Higher values (4.23 g·100 g^–1^) have been reported by Aremu et al.^83^ in studies on flamboyant-mirim seed flour.

Betaine showed higher concentrations in the aqueous extract (0.441 mg·100 mg^–1^) and lower concentrations in the methanolic extract (0.172 mg·100 mg^–1^). Cysteine was also more abundant in the aqueous extract, with a value of 1.361 mg·100 mg^–1^, compared with 0.228 mg·100 mg^–1^ in the methanolic extract. Alanine was detected only in the aqueous FSF extract at a concentration of 0.216 mg·100 mg^–1^. Higher alanine levels (4.09 g·100 g^–1^) were previously reported by Aremu et al.^83^ in flamboyant-mirim seed flour.

Sucrose exhibited the highest concentration among all identified compounds, with 20.095 mg·100 mg^–1^ in the aqueous extract and 4.227 mg·100 mg^–1^ in the methanolic extract. Galactose and mannose were identified exclusively in the aqueous extract, at concentrations of 2.646 mg·100 mg^–1^ and 1.953 mg·100 mg^–1^, respectively. According to da Cunha Jácome Marques et al.,^3^ the galactomannan extracted from *Caesalpinia pulcherrima* seeds contains approximately 63.4% mannose and 29.1% galactose, corroborating the presence of these monosaccharides in the aqueous extract.

Citric acid was also detected in the aqueous extract, with a concentration of 1.729 mg·100 mg^–1^, indicating the coexistence of organic acids that may contribute to the acidity and potential antioxidant activity of the FSF extracts.

### 3.6 Multivariate Statistical Analysis

A supervised multivariate statistical analysis using partial least-squares–discriminant analysis (PLS-DA) was performed to investigate the variability of 28 variables in flamboyant-mirim seed flour (FSF) samples according to their particle sizes (0.250, 0.355, 0.500, and 0.710 mm). Figure 3a presents the LV1 × LV2 scores plot; Figure 3b shows the Hotelling’s *T* ^2^ versus *Q*-residuals plot; Figure 3c illustrates the LV1 × LV2 loadings plot; and Figure 3d displays the variable importance in projection (VIP) results.

**Figure 3:**
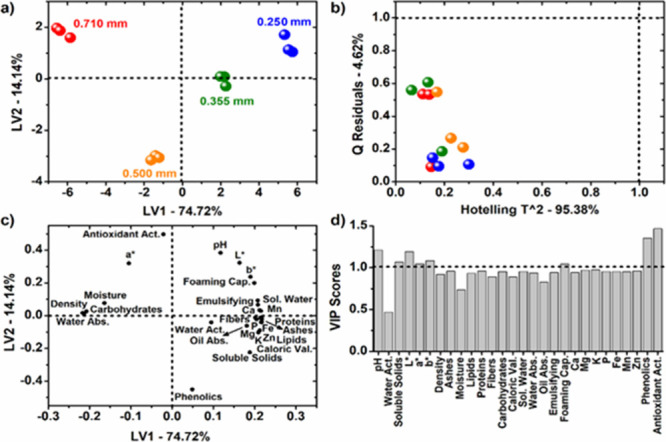
PLS-DA results for the classification of flamboyant-mirim (*Caesalpinia pulcherrima* (L.) Swartz) seed flour samples according to particle size: (a) scores plot (LV1 × LV2); (b) Hotelling’s *T* ^2^ vs *Q*-residuals; (c) loadings plot (LV1 × LV2); and (d) VIP (variable importance in projection).

As illustrated in Figure 3a, clear clustering trends were observed in the scores plot, with the particle size increasing from positive to negative LV1 scores. According to the loading plot (Figure 3c), higher particle sizes were associated with higher bulk density, moisture, carbohydrates, water absorption capacity, and a*. For the phenolic/antioxidant descriptors, it is important to note that the behavior across fractions was not strictly monotonic (Table 4), and the antioxidant variable included in the PLS-DA corresponds to EC_50_ (DPPH) rather than “activity” per se. Therefore, higher EC_5_0 values indicate a lower antioxidant performance. Conversely, increasing particle size was related to lower values of pH, water activity, soluble solids, L*, b*, ash, lipids, proteins, fiber, caloric value, water solubility index, oil absorption capacity, emulsifying activity, foaming capacity, and the minerals calcium, magnesium, potassium, phosphorus, iron, manganese, and zinc.

In particular, the LV2 axis indicated that among the higher particle size flours (0.500 and 0.710 mm, with negative LV1 scores), the 0.710 mm FSF fraction exhibited higher EC_50_ values (corresponding to lower antioxidant potential) than the 0.500 mm FSF fraction, whereas the latter showed greater total phenolic content.

Moreover, the VIP results revealed that variations in pH, soluble solids, L*, a*, b*, foaming capacity, total phenolics, and antioxidant capacity were the most relevant variables for discriminating among the FSF fractions, in agreement with chemometric criteria commonly adopted in food systems.^1–3^ The Hotelling’s *T* ^2^ versus *Q*-residual plot (Figure 3b) indicated that no samples negatively influenced the model fitting, as none exceeded the threshold values, confirming the robustness of the PCA model.^84^ These multivariate patterns corroborate the univariate results (Tables 1–4) and are consistent with previous applications of PCA and VIP-based interpretation in cereal flour systems.^85^

Tables 1–4 corroborated and complemented the variability patterns identified through multivariate classification modeling. Additionally, ANOVA results confirmed the statistical significance of the variations in pH, soluble solids, L*, a*, b*, foaming capacity, total phenolics, and antioxidant capacity highlighted by the VIP analysis.

## 4 Conclusions

The FSF fraction with a particle size of 0.250 mm exhibited the highest contents of ash, lipids, proteins, fibers, and caloric value. This finer fraction also showed superior levels of all analyzed minerals and remarkable technological properties, including a higher water solubility index, oil absorption capacity, foaming capacity, and emulsifying activity. The 0.500 mm fraction, in turn, was distinguished by its higher total phenolic content and antioxidant performance compared to the other particle sizes.

The NMR results detailed the composition variability of the flamboyant-mirim seed flour according to the type of solvent (chloroform, methanol, and water), and it was able to quantify the organic compounds with nonoverlapped signals, providing simultaneous structural and quantitative results with high accuracy without needing analyte-specific standards, which is suitable for complex mixtures without the necessity of sample purification or compound isolation. Therefore, the NMR analysis revealed that the composition and concentration of compounds varied according to the solvent used for extraction, highlighting differences in the chemical profiles among the extracts. Multivariate statistical modeling by PLS-DA confirmed that pH, soluble solids, color parameters (L*, a*, b*), foaming capacity, total phenolics, and antioxidant activity were the most discriminating variables among the FSF fractions.

Overall, the results demonstrate that FSF fractions, particularly those with smaller particle sizes, possess valuable nutritional, functional, and technological attributes, supporting their potential application as multifunctional ingredients in diverse food formulations.

## References

[1] Mariutti L. R. B., Rebelo K. S., Bisconsin-Junior A., de Morais J. S., Magnani M., Maldonade I. R., Madeira N. R., Tiengo A., Maróstica M. R., Cazarin C. B. B. (2021). The Use of Alternative Food Sources
to Improve Health and Guarantee Access and Food Intake. Food Res. Int..

[2] Carvalho P. O. A. A., Guerra G. C. B., Borges G. d. S. C., Bezerril F. F., Sampaio K. B., Ribeiro T. S., Pacheco M. T. B., Milani R. F., Goldbeck R., Ávila P. F., Lima M. d. S., Souza M. d. F. V., Queiroga R. d. C. R. d. E. (2021). Nutritional
Potential and Bioactive
Compounds of Xique-Xique Juice: An Unconventional Food Plant from
Semiarid Brazilian. J. Food Process. Preserv..

[3] da
Cunha Jácome Marques F., da Silva Pantoja P., Matos V. E. A., Silva R. O., Damasceno S. R. B., Franco Á. X., Alves R. C., Justino P. F. C., de Souza M. H. L. P., Feitosa J. P. A., Castro R. R., Soares P. M. G. (2019). Galactomannan from the Seeds of *Caesalpinia
pulcherrima* Prevents Indomethacin-Induced Gastrointestinal
Damage via Neutrophil Migration. Int. J. Biol.
Macromol..

[4] Egounlety M., Aworh O. C. (2003). Effect of soaking,
dehulling, cooking and fermentation
with *Rhizopus oligosporus* on the oligosaccharides,
trypsin inhibitor, phytic acid and tannins of soybean (*Glycine
max* Merr.), cowpea (*Vigna unguiculata* L.
Walp) and groundbean (*Macrotyloma geocarpa* Harms). J. Food Eng..

[5] Pal R. S., Bhartiya A., Yadav P. (2017). Effect of dehulling,
germination and cooking on nutrients, anti-nutrients, fatty acid composition
and antioxidant properties in lentil (*Lens culinaris*). J. Food Sci. Technol..

[6] Cheng S., Langrish T. A. G. (2025). A Review of the
Treatments to Reduce Anti-Nutritional
Factors and Fluidized Bed Drying of Pulses. Foods.

[7] Zhong L., Fang Z., Wahlqvist M. L., Wu G., Hodgson J. M., Johnson S. K. (2018). Seed coats of pulses as a food ingredient: Characterization,
processing, and applications. Trends Food Sci.
Technol..

[8] Yusuf A. A., Mofio B. M., Ahmed A. B. (2007). Nutrient Contents of Prides of Barbados
(*Caesalpinia pulcherrima* Linn) Seeds. Pak. J. Nutr..

[9] Chanda S., Parekh J., Baravalia Y., Parekh S. (2010). Antimicrobial and Antioxidant
Efficacy of Various Solvent Extracts of Seeds and Fruit Rind of *Caesalpinia pulcherrima* Swartz. Arch.
Clin. Microbiol..

[10] Roumy V., Ruiz L., Ruiz Macedo J. C., Gutierrez-Choquevilca A. L., Samaillie J., Encinas L. A., Mesia W. R., Ricopa
Cotrina H. E., Rivière C., Sahpaz S., Bordage S., Garçon G., Dubuisson J., Anthérieu S., Seron K., Hennebelle T. (2020). Viral Hepatitis in the Peruvian Amazon:
Ethnomedical Context and Phytomedical Resource. J. Ethnopharmacol..

[11] FALOYE K. O., FAMUYIWA S. O., AKINWUNMI K. F., TATA C. M., AYOOLA M. D., FAKOLA E. G., AKINYELE O. F., NDINTEH D. T. (2022). Antioxidant Potentials
of the Pod Extract of *Caesalpinia pulcherrima* Swartz
(Fabaceae) and the Theoretical Evaluation of the Antioxidant Property
of the Isolated Compounds. Int. J. Sci. Res.
Arch..

[12] Erharuyi O., Adhikari A., Falodun A., Jabeen A., Imad R., Ammad M., Choudhary M. I., Gören N. (2017). Cytotoxic,
Anti-inflammatory, and Leishmanicidal Activities of Diterpenes Isolated
from the Roots of *Caesalpinia pulcherrima*. Planta Med..

[13] Sharma V., Rajani G. P. (2011). Evaluation of *Caesalpinia pulcherrima* Linn. for Anti-inflammatory and Antiulcer Activities. Indian J. Pharmacol..

[14] Kumar D., Singh J., Baghotia A., Kumar S. (2010). Anticonvulsant Effect
of the Ethanol Extract of *Caesalpinia pulcherrima* (L.) Sw., Fabaceae, Leaves. Braz. J. Pharmacogn..

[15] Singh G., Suttee A., Barnwal R. P., Singla N., Sharma A., Chatterjee M., Kaura G., Chanana V., Mishra V. K. (2019). Investigation
of *In Vitro* Anthelmintic Activity of *Caesalpinia
pulcherrima* Leaves. Plant Arch..

[16] Oderinde R.
A., Adewuyi A., Ajayi I. A. (2008). Determination of the Mineral Nutrients,
Characterization and Analysis of the Fat-Soluble Vitamins of *Caesalpinia pulcherrima* and *Albizia lebbeck* Seeds and Seed Oils. Seed Sci. Biotechnol..

[17] Buriti F.
C. A., dos Santos K. M. O., Sombra V. G., Maciel J. S., Teixeira Sá D. M. A., Salles H. O., Oliveira G., de Paula R. C. M., Feitosa J. P. A., Monteiro Moreira A. C. O., Moreira R. A., Egito A. S. (2014). Characterisation
of Partially Hydrolysed
Galactomannan from *Caesalpinia pulcherrima* Seeds
as a Potential Dietary Fibre. Food Hydrocoll..

[18] Senarathna S., Navaratne S., Wickramasinghe I., Coorey R. (2022). Development and Characterization
of *Caesalpinia pulcherrima* Seed Gum-Based Films to
Determine Their Applicability in Food Packaging. J. Consum. Prot. Food Saf..

[19] Passos A. A. C., Teixeira-Sá D.
M. A., Morais G. M. D., Chacon L. S. S., Braga R. C. (2016). Avaliação da Incorporação
de Galactomana de *Caesalpinia pulcherrima* em Sorvetes
e Comparação com Estabilizantes Comerciais. Rev. Ciênc. Agron..

[20] Medeiros S. R. A., Oliveira V. A. d., Oliveira A. M. C. d., Araujo M. L. H., Feitosa J. P. d. A., Paula R. C. M. d., Sousa F. D. d., Moreira A. C. d. O. M., Beserra F. J., Moreira R. d. A. (2020). *Caesalpinia pulcherrima* Seed Galactomannan on Rheological
Properties of Dairy Desserts. Ciênc.
Rural.

[21] Bhagya N. (2024). A Critical
Review on the Phytochemistry, Pharmacology and Toxicology of *Caesalpinia pulcherrima* (L.) Sw. S.
Afr. J. Bot..

[22] Agbede J. O. (2004). Compositional
Studies of Differently Processed Ornamental Plant Seed Flour: *Caesalpinia pulcherima*. Pak. J. Nutr..

[23] Rudakova T., Minorova A., Moiseeva L., Krushelnytska N., Narizhnyy S., Osipenko I., Bovkun A. (2024). Study of structural
and mechanical characteristics of dairy desserts with a combined composition
of raw materials. Anim. Husb. Prod. Prod. Process..

[24] González-Montemayor A. M., Flores-Gallegos A. C., Contreras-Esquivel J.
C., Solanilla-Duque J. F., Rodríguez-Herrera R. (2019). *Prosopis* spp. Functional
Activities and Its Applications in Bakery Products. Trends Food Sci. Technol..

[25] de
Gusmão R. P., Cavalcanti-Mata M. E.
R. M., Duarte M. E. M., Gusmão T. A. S. (2016). Particle Size, Morphological, Rheological,
Physicochemical Characterization and Designation of Minerals in Mesquite
Flour (*Prosopis juliflora*). J. Cereal Sci..

[26] Nabil B., Ouaabou R., Ouhammou M., Saadouni L., Mahrouz M. (2020). Impact of
Particle Size on Functional, Physicochemical Properties and Antioxidant
Activity of Cladode Powder (*Opuntia ficus-indica*). J. Food Sci. Technol..

[27] Bala M., Handa S., D M., Singh R. K. (2020). Physicochemical,
Functional, and Rheological Properties of Grass Pea (*Lathyrus
sativus* L.) Flour as Influenced by Particle Size. Heliyon.

[28] American Association of Cereal Chemists . Approved Methods of the American Association of Cereal Chemists, 1th ed.; American Association of Cereal Chemists Paul, MN, USA, 2000; Vol. 2.

[29] Association of Official Analytical Chemists . Official Methods of Analysis, 1th ed.; AOAC: Arlington, VA, USA, 2005.

[30] Kaur M., Sandhu K. S., Arora A., Sharma A. (2015). Gluten-Free Biscuits
Prepared from Buckwheat Flour by Incorporation of Various Gums: Physicochemical
and Sensory Properties. *LWT–Food*. Sci. Technol..

[31] Du S. K., Jiang H., Yu X., Jane J. L. (2014). Physicochemical
and Functional Properties of Whole Legume Flour. *LWT–Food*. Sci. Technol..

[32] Kaur M., Singh N. (2005). Studies on Functional,
Thermal and Pasting Properties of Flours from
Different Chickpea (*Cicer arietinum* L.) Cultivars. Food Chem..

[33] Silva, D. J. ; Queiroz, A. C. Análise de Alimentos: Métodos Químicos e Biológicos, 3. ed.; UFV: Viçosa, MG, Brazil, 2006.

[34] Swain T., Hillis W. E. (1959). The Phenolic Constituents of *Prunus domestica*. I. Quantitative Analysis of Phenolic Constituents. J. Sci. Food Agric..

[35] Vieira L. M., Sousa M. S. B., Mancini-Filho J., Lima A. (2011). Fenólicos Totais
e Capacidade Antioxidante *In Vitro* de Polpas de Frutos
Tropicais. Rev. Bras. Frutic..

[36] Brand-Williams W., Cuvelier M. E., Berset C. (1995). Use of a Free
Radical Method to Evaluate
Antioxidant Activity. *LWT–Food*. Sci. Technol..

[37] Pauli G. F., Jaki B. U., Lankin D. C. (2005). Quantitative
1H NMR: Development
and Potential of a Method for Natural Products Analysis. J. Nat. Prod..

[38] Alves
Filho E. G., Silva L. M. A., Teofilo E. M., Larsen F. H., de Brito E. S. (2017). ^1^H NMR Spectra Dataset and Solid-State NMR
Data of Cowpea (*Vigna unguiculata*). Data Brief.

[39] Cheng F., Ding K., Yin H., Tulbek M., Fabek H., Chigwedere C. M., Anderson H., Ai Y. (2025). Influence of coarse
particles on nutritional quality of flour ingredients: A comparison
between cereals and pulses. J. Food Compos.
Anal..

[40] Assatory A., Vitelli M., Rajabzadeh A. R., Legge R. L. (2019). Dry fractionation
methods for plant protein, starch and fiber enrichment: A review. Trends Food Sci. Technol..

[41] Adewuyi A., Oderinde R. A. (2014). Fatty Acid Composition
and Lipid Profile of *Diospyros mespiliformis*, *Albizia lebbeck*, and *Caesalpinia pulcherrima* Seed Oils from Nigeria. Int. J. Food Sci..

[42] Mu Y., Yan J., Gao J., Xing F., Gu Q., Zhao C., Liu J. (2025). Processing
properties of corn flour and quality of fresh wet corn–wheat
noodles: Comparison of ultrasonic-assisted nixtamalization wet milling
with traditional milling methods. LWT.

[43] Rivera J., Siliveru K., Li Y. (2022). A comprehensive
review on pulse protein
fractionation and extraction: processes, functionality, and food applications. Crit. Rev. Food Sci. Nutr..

[44] Fernando S. (2021). Production
of protein-rich pulse ingredients through dry fractionation: A review. LWT.

[45] Soncin
Alfaro G. M., McGee R. J., Kiszonas A. M. (2025). Influence of genotype
and environment on field pea composition and milling traits. J. Sci. Food Agric..

[46] Chávez-Mendoza C., Hernández-Figueroa K. I., Sánchez E. (2019). Antioxidant
capacity and phytonutrient content in the seed coat and cotyledon
of common beans (*Phaseolus vulgaris* L.) from various
regions in Mexico. Antioxidants (Basel).

[47] Gouseti O., Lovegrove A., Kosik O., Fryer P. J., Mills C., Gates F., Tucker G., Latty C., Shewry P., Bakalis S. (2019). Exploring
the Role of Cereal Dietary Fiber in Digestion. J. Agric. Food Chem..

[48] Savlak N., Türker B., Yeşilkanat N. (2016). Effects of Particle Size Distribution
on Some Physical, Chemical and Functional Properties of Unripe Banana
Flour. Food Chem..

[49] Nabil B., Ouaabou R., Ouhammou M., Essaadouni L., Mahrouz M. (2020). Functional Properties, Antioxidant
Activity, and Organoleptic
Quality of Novel Biscuit Produced by Moroccan Cladode Flour “*Opuntia ficus-indica*”. J. Food
Qual..

[50] Franco, B. D. G. ; Landgraf, M. Microbiologia dos Alimentos; Atheneu: Rio de Janeiro, Brazil, 2004.

[51] Ahmed J., Al-Jassar S., Thomas L. (2015). A Comparison in Rheological, Thermal,
and Structural Properties between Indian Basmati and Egyptian Giza
Rice Flour Dispersions as Influenced by Particle Size. Food Hydrocoll..

[52] Miganeh
Waiss I., Kimbonguila A., Mohamed Abdoul-Latif F., Nkeletela L. B., Scher J., Petit J. (2021). Total Phenolic Content,
Antioxidant Activity, Shelf-Life and Reconstitutability of Okra Seeds
Powder: Influence of Milling and Sieving Processes. Int. J. Food Sci. Technol..

[53] Jima B. R., Abera A. A., Kuyu C. G. (2025). Effect of particle size on compositional,
functional, pasting, and rheological properties of teff *Eragrostisteff
(Zucc.)*. Trotterflour. Appl. Food Res..

[54] Wang Q., Li L., Zheng X. (2020). A review of
milling damaged starch: Generation, measurement,
functionality and its effect on starch-based food systems. Food Chem..

[55] Teobaldi A. G. (2025). The Properties of Damaged
Starch Granules: The Relationship Between
Granule Structure and Water–Starch Polymer Interactions. Foods.

[56] Islam M. A. (2024). Particle size reduction influences starch and protein functionality,
and nutritional quality of stone milled whole wheat flour from hard
red spring wheat. Food Biosci..

[57] Pinheiro G. K. I., Oliveira D. E. C., Resende O., Junior W. N. F., Cabral J. C. O., Quequeto W. D., Silva L. C. M., Souza D. G. (2022). Physical,
Physicochemical and Functional Technological Properties of Flours
Produced from Yellow Mombin (*Spondias mombin* L.)
Epicarp. Aust. J. Crop Sci..

[58] Jiang L., Xu Q. X., Qiao M., Ma F. F., Thakur K., Wei Z. J. (2017). Effect of Superfine
Grinding on Properties of *Vaccinium bracteatum* Thunb
Leaves Powder. Food Sci. Biotechnol..

[59] Chandra S., Singh S., Kumari D. (2014). Evaluation of Functional Properties
of Composite Flours and Sensorial Attributes of Composite Flour Biscuits. J. Food Sci. Technol..

[60] Sahu P. K., Cervera-Mata A., Chakradhari S., Singh Patel K., Towett E. K., Quesada-Granados J. J., Martín-Ramos P., Rufián-Henares J. A. (2022). Seeds as Potential
Sources of Phenolic
Compounds and Minerals for the Indian Population. Molecules.

[61] Omode A. A., Fatoki O. S., Olaogun K. A. (1995). Physicochemical
Properties of Some
Underexploited and Nonconventional Oilseeds. J. Agric. Food Chem..

[62] Ahmed J., Thomas L., Arfat Y. A. (2019). Functional, Rheological, Microstructural,
and Antioxidant Properties of Quinoa Flour in Dispersions as Influenced
by Particle Size. Food Res. Int..

[63] Kim J.-M., Shin M. (2014). Effects of particle
size distributions of rice flour on the quality
of gluten-free rice cupcakes. LWT - Food Sci.
Technol..

[64] Chiodetti M., Tuccio M. G., Carini E. (2024). Effect of water content
on gelatinization
functionality of flour from sprouted sorghum. Curr. Res. Food Sci..

[65] Brasil, ANVISA . Ministério da Saúde. Agência Nacional de Vigilância Sanitária. Resolução n° 711, de 1° de Julho de 2022. Dispõe sobre o Regulamento Técnico para Produtos de Cereais, Amidos, Farinhas e Farelos; Diário Oficial da República Federativa do Brasil 2022.

[66] Sweers L. J. H., Politiek R. G. A., Lakemond C. M. M., Bruins M. E., Boom R. M., Fogliano V., Mishyna M., Keppler J. K., Schutyser M. A. I. (2022). Dry fractionation for protein enrichment
of animal
by-products and insects: A review. J. Food Eng..

[67] Chinma C. E., Abu J. O., Asikwe B. N., Sunday T., Adebo O. A. (2021). Effect
of Germination on the Physicochemical, Nutritional, Functional, Thermal
Properties and *In Vitro* Digestibility of Bambara
Groundnut Flours. LWT–Food Sci. Technol..

[68] Memon A. A., Mahar I., Memon R., Soomro S., Harnly J., Memon J., Bhangar M. I., Luthria D. L. (2020). Impact of Flour
Particle Size on Nutrient and Phenolic Acid Composition of Commercial
Wheat Varieties. J. Food Compos. Anal..

[69] Ramondo A., Marulo S., Sorrentino A., Masi P., Di Pierro P. (2024). Modification
of Physicochemical and Functional Properties of Pumpkin Seeds Protein
Isolate (PsPI) by High-Intensity Ultrasound: Effect of Treatment Time. ACS Food Sci. Technol..

[70] Destribats M., Eyharts M., Lapeyre V., Sellier E., Varga I., Ravaine V., Schmitt V. (2014). Impact of
pNIPAM Microgel Size on
Its Ability to Stabilize Pickering Emulsions. Langmuir.

[71] Xiong X., Ho M. T., Bhandari B., Bansal N. (2020). Foaming properties
of milk protein dispersions at different protein content and casein
to whey protein ratios. Int. Dairy J..

[72] Rempel C., Geng X., Zhang Y. (2019). Industrial
scale preparation of pea
flour fractions with enhanced nutritive composition by dry fractionation. Food Chem..

[73] Saldanha
do Carmo C., Silventoinen P., Nordgård C. T., Poudroux C., Dessev T., Zobel H., Holtekjølen A. K., Draget K. I., Holopainen-Mantila U., Knutsen S. H., Sahlstrøm S. (2020). Is dehulling
of peas and faba beans necessary prior to dry fractionation for the
production of protein- and starch-rich fractions? Impact on physical
properties, chemical composition and techno-functional properties. J. Food Eng..

[74] Thakur S., Scanlon M. G., Tyler R. T., Milani A., Paliwal J. (2019). Pulse Flour
Characteristics from a Wheat Flour Miller’s Perspective: A
Comprehensive Review. Compr. Rev. Food Sci.
Food Saf..

[75] Hendek-Ertop M., Bektaş M., Atasoy R. (2020). Effect of Cereals Milling on the
Contents of Phytic Acid and Digestibility of Minerals and Protein. Ukrainian Food J..

[76] Dela
Torre G. L. T. (2017). Evaluation of Antioxidant Capacity and Identification
of Bioactive Compounds of Crude Methanol Extracts of *Caesalpinia
pulcherrima* (L.) Swartz. Indian J.
Pharm. Sci..

[77] Becker L., Zaiter A., Petit J., Zimmer D., Karam M. C., Baudelaire E., Scher J., Dicko A. (2016). Improvement of Antioxidant
Activity and Polyphenol Content of *Hypericum perforatum* and *Achillea millefolium* Powders Using Successive
Grinding and Sieving. Ind. Crops Prod..

[78] Alves M. J., Moura A. K. S., Costa L. M., Araújo E. J. F., Sousa G. M., Costa N. D. J., Ferreira M. P. M., Silva J. N., Pessoa C., Lima S. G., Citó A. M. G. L. (2014). Teor
de Fenóis e Flavonoides, Atividades Antioxidante e Citotóxica
das Folhas, Frutos, Cascas dos Frutos e Sementes de *Piptadenia
moniliformis* Benth (*Leguminosae – Mimosoideae*). Bol. Latinoam. Caribe Plantas Med. Aromat..

[79] Azeem M., Mu T., Zhang M. (2021). Influence
of Particle Size Distribution on Nutritional
Composition, Microstructural, and Antioxidant Properties of Orange
and Purple-Fleshed Sweet Potato Flour. J. Food
Process. Preserv..

[80] Oyeyinka S. A., Adepegba A. A., Oyetunde T. T., Oyeyinka A. T., Olaniran A. F., Iranloye Y. M., Olagunju O. F., Manley M., Kayitesi E., Njobeh P. B. (2021). Chemical, Antioxidant
and Sensory Properties of Pasta
from Fractionated Whole Wheat and Bambara Groundnut Flour. LWT–Food Sci. Technol..

[81] Singh B., Singh J. P., Kaur A., Singh N. (2017). Phenolic Composition
and Antioxidant Potential of Grain Legume Seeds: A Review. Food Res. Int..

[82] Deli M., Ndjantou E. B., Ngatchic Metsagang J.
T., Petit J., Njintang Yanou N., Scher J. (2019). Successive Grinding and Sieving as
a New Tool to Fractionate Polyphenols and Antioxidants of Plant Powders:
Application to *Boscia senegalensis* Seeds, *Dichrostachys glomerata* Fruits, and *Hibiscus sabdariffa* Calyx Powders. Food Sci. Nutr..

[83] Aremu M. O., Bamidele T. O., Nweze C. C., Idris I. M. (2012). Chemical Evaluation
of Prides of Barbados (*Caesalpinia pulcherrima*) Seeds
Grown in Gudi, Nasarawa State. Nigeria. Int.
J. Chem. Sci..

[84] Bevilacqua, M. ; Bucci, R. ; Magrì, A. D. ; Magrì, A. L. ; Marini, R. N. F. In Chemometrics in Food Chemistry; Marini, F. , Ed.; Elsevier: Oxford, U.K., 2013; p 498.

[85] Jima B. R., Abera A. A., Kuyu C. G. (2025). Effect
of particle size on compositional,
functional, pasting, and rheological properties of teff flour. Appl. Food Res..

